# In-vitro alpha amylase inhibitory activity of the leaf extracts of *Adenanthera pavonina*

**DOI:** 10.1186/s12906-016-1452-y

**Published:** 2016-11-15

**Authors:** M. Nirmali Wickramaratne, J. C. Punchihewa, D. B. M. Wickramaratne

**Affiliations:** 1 Department of Physical Sciences and Technology, Faculty of Applied Sciences, Sabaragamuwa University of Sri Lanka, Belihuloya, Sri Lanka; 2 Department of Pharmacy Faculty of Allied Health Sciences, University of Peradeniya, Peradeniya, Sri Lanka; 3 Faculty of Allied Health Sciences, University of Peradeniya, Peradeniya, Sri Lanka

## Abstract

**Background:**

Diabetes has caused a major burden to the health sector in the developing countries and has shown an increasing trend among the urban population. It is estimated that most patients are with type II diabetes which could be easily treated with dietary changes, exercise, and medication. Sri Lanka carries a long history ayurvedic medicine where it uses the plant for treating many diseases. Therefore it is important to screen medicinal plants scientifically so they could be used safely and effectively in the traditional medical system and also be used for further investigations.

*Adenanthera pavonina* is a plant used in the Ayurvedic medical system in Sri Lanka for treating many diseases including diabetics. We evaluated the anti-diabetic properties and the antioxidant properties of *Adenanthera pavonina* leaves.

**Methods:**

The methanol extract of the leaves was sequentially extracted with petroleum ether and thereafter was partitioned between EtOAc, and water. The α-amylase inhibition assay was performed using the 3,5- dinitrosalicylic acid method. The antioxidant activities were measured using the DPPH free radical scavenging activity and the total phenolic content using Folin-Ciocalteu’s reagent. The cytotoxicity of the extract was evaluated using the Brine shrimp bioassay.

**Results:**

The IC_50_ values of α amylase inhibitory activity of MeOH, EtOAc, petroleum ether, and water were 16.16 ± 2.23, 59.93 ± 0.25, 145.49 ± 4.86 and 214.85 ± 9.72 μg/ml respectively and was similar to that of Acarbose (18.63 ± 1.21 (μg/ml). Antioxidant activities were also determined and the EtOAc fraction showed the highest total phenolic content (34. 62 ± 1.14 mg/g extract) and the highest DPPH scavenging activity with an IC_50_ of 249.92 ± 3.35 μg/ml.

**Conclusion:**

The leaf extracts of *Adenanthera pavonina* exhibit remarkable α-amylase inhibitory activity in the crude methanolic extract. Hence leaves of *Adenanthera pavonina* has a potential to be used as a regular green vegetable and also be investigated further in isolating pure compounds with anti-diabetic activity.

## Background

It is estimated that the global prevalence of diabetes is increasing each year causing a major burden to the health sector, especially in the developing countries. It is estimated that the prevalence of diabetes is high among the urban population [1] where 90% is accounted to be type II diabetic [2]. Patients with type II diabetes can be easily treated with dietary changes, exercise, and medication. The complications arising with diabetes are closely related to the production of free radicals enhancing the oxidative stress [3]. Hence the use of antioxidants has been effective in reducing the severity of diabetic complications [4].

The traditional medical systems in countries like China, India, North Africa and Sri Lanka use decoctions or fresh mixtures of juices made from medicinal plants in treating most diseases like cancer, arthritis, diabetics etc. *Adenanthera pavonina* (Madatiya –Sinhala, Circassian bean–English) belonging to the family Fabaceae; used in traditional herbal medicine for a variety of diseases including diabetes, lipid disorders, diarrhea, hemorrhage, and as an anti-inflammatory agent [5]. Phytochemical investigations of the plant have identified many compounds which are steroids, glycosides, alkaloids, saponins, polysaccharides, fatty acids and various amino acids [5, 6] with no claims of any of the above compounds to be active as an antidiabetic compound. Studies performed on the seed and leaf extracts on rat models have shown to possess hypoglycemic and antihyperglycemic activity [7]. The seeds are reported to be toxic, but some consume the roasted seed while the suburban population in countries like India and Sri Lanka, consumes the tender leaves as a green vegetable in their regular diet (http://www.worldagroforestry.org/treedb/AFTPDFS/Adenanthera_pavonina.PDF).

It is important to evaluate the antidiabetic activity of the leaves quantitatively which would allow the use of *Adenanthera pavonina* (*A. pavonina*) leaves in a more efficient and effective manner in treating diabetics. Therefore the present investigation was carried out to evaluate the in-vitro α - amylase inhibitory activity and the antioxidant activity of the leaf extracts of *A. pavonina*.

## Methods

All chemicals used were of analytical reagent grade. 1,1-Diphenyl-2-picrylhydrazyl radical (DPPH), 3,5 dinitro salicylic acid (DNSA), Gallic acid and α-amylase from *Aspergillus oryzae* purchased from (Sigma USA). Dimethylsulfoxide (DMSO) was purchased Sigma-Aldrich. UV-visible spectrophotometer (Thermo Fisher Scientific G10S).

Collection of plant materials: Fresh leaves of *A. pavonina* were collected from Peradeniya and their authenticity was confirmed by The National Herbarium, Royal Botanic Gardens, Peradeniya, Sri Lanka.

### Plant extract

Air-dried, powdered leaves (200 g) of *A. pavonina* were extracted with methanol (MeOH) at room temperature for 2 × 24 h using 1500 ml of MeOH for each extraction. The combined MeOH extracts were concentrated under vacuum at 40 °C to 1 L and were partitioned into petroleum ether (2 × 500 ml). The combined petroleum ether fraction was filtered and dried over anhydrous Na_2_SO_4_ and was evaporated under vacuum to dryness. The dried petroleum ether extract was refrigerated in a tightly closed container until use. The remaining methanol fraction was evaporated under vacuum to dryness and residue was partitioned between ethyl acetate (EtOAc, 1 L) and water (1 L). The ethyl acetate extract was dried over anhydrous Na_2_SO_4_ and was evaporated under vacuum to dryness and refrigerated until further use. The aqueous fraction was frozen and freeze-dried and the residue was stored in the refrigerator until further use.

### In vitro α-amylase inhibitory studies

The α-amylase inhibition assay was performed using the 3,5-dinitrosalicylic acid (DNSA) method [8]. The leaf extract of *A. pavonina* was dissolved in minimum amount of 10% DMSO and was further dissolved in buffer ((Na_2_HPO_4_/NaH_2_PO_4_ (0.02 M), NaCl (0.006 M) at pH 6.9) to give concentrations ranging from 10 to 1000 μg/ml. A volume of 200 μl of α-amylase solution (2 units/ml) was mixed with 200 μl of the extract and was incubated for 10 min at 30 °C. Thereafter 200 μl of the starch solution (1% in water (w/v)) was added to each tube and incubated for 3 min. The reaction was terminated by the addition of 200 μl DNSA reagent (12 g of sodium potassium tartrate tetrahydrate in 8.0 mL of 2 M NaOH and 20 mL of 96 mM of 3,5-dinitrosalicylic acid solution) and was boiled for 10 min in a water bath at 85–90 °C. The mixture was cooled to ambient temperature and was diluted with 5 ml of distilled water, and the absorbance was measured at 540 nm using a UV-Visible spectrophotometer. The blank with 100% enzyme activity was prepared by replacing the plant extract with 200 μl of buffer. A blank reaction was similarly prepared using the plant extract at each concentration in the absence of the enzyme solution. A positive control sample was prepared using Acarbose (100 μg/ml–2 μg/ml) and the reaction was performed similarly to the reaction with plant extract as mentioned above. The α-amylase inhibitory activity was expressed as percent inhibition and was calculated using the equation given below: The % α-amylase inhibition was plotted against the extract concentration and the IC_50_values were obtained from the graph.

$$ \%\kern0.5em \alpha \kern0.5em \mathrm{amylase}\kern0.5em \mathrm{inhibition}\kern0.5em =\kern0.5em 100\times \frac{\mathrm{Ab}{\mathrm{s}}_{100\%\kern0.5em \mathrm{control}}-\mathrm{Ab}{\mathrm{s}}_{\mathrm{Sample}}}{\mathrm{Ab}{\mathrm{s}}_{100\%\kern0.5em \mathrm{Control}}} $$

### Antioxidant activity of *A. pavonina* leaf extract

#### DPPH radical scavenging activity

Free radical scavenging activity of *A. pavonina* leaf extract was measured using the DPPH assay as in Gulluce et al., [9] with slight modifications. The plant extract was dissolved in methanol and prepared samples with different concentrations ranging from 50 to 1000 μg/ml. A methanolic solution (0.1 M) of DPPH was freshly prepared and kept in dark at 4 °C until use. A volume of 1 ml of the extract was added into 1 ml of methanolic DPPH solution. Replacing the extract with 100% methanol solution the control reaction was made. The mixture was incubated for 30 min in dark at ambient temperature and thereafter the absorbance was measured at 517 nm UV-Visible spectrophotometer [10]. The Antioxidant activity was calculated using the following equation:

$$ \%\kern0.5em \mathrm{DPPH}\kern0.5em \mathrm{Scavenging}\kern0.5em \mathrm{activity}\kern0.5em =\kern0.5em 100\times \frac{\mathrm{Ab}{\mathrm{s}}_{\mathrm{Control}}\hbox{-} \mathrm{Ab}{\mathrm{s}}_{\mathrm{Sample}}}{\mathrm{Ab}{\mathrm{s}}_{\mathrm{Control}}} $$

### Total phenolic content

The total phenolic content of *A.pavonina* was determined spectrophotometrically using Folin-Ciocalteu’s reagent. The extract (100 μl ;1 mg/ml) was mixed with 100 μl of Folin-Ciocalteu’s phenol reagent and kept for 5 min. A volume of 1.00 ml of a 7% Na_2_CO_3_ solution was added to the reaction mixture followed by addition of 1.30 ml of deionized (DI) water. The mixture was kept in the dark at ambient temperature for 90 min and thereafter the absorbance measured at 750 nm. A calibration graph was prepared using Gallic acid as the standard and the total phenolic content in the extract was expressed as milligrams of Gallic acid equivalents in one gram of extract [10, 11].

### Brine shrimp cytotoxicity bioassay

The plant extract was dissolved initially in 100 μl of DMSO and was further dissolved in artificial sea water to give concentrations ranging from 125 to 1000 μg/ml. Artificial sea water was prepared using the Cold Spring Harbor Laboratory protocol, 2011 [12]. A 26.29 g of NaCl (450 mM), 0.74 g KCl (10 mM), CaCl_2_ 0.99 g (9 mM), MgCl_2_•6H_2_O 6.09 g (30 mM) and MgSO_4_•7H_2_O 3.94 g (16 mM) was dissolved in 1 L deionized water and was filtered by using Whatmann filter paper No. 42. The pH of the solution was adjusted to 7.8 and autoclaved and was store at 4 °C until use (maximum for 1 week). Two milliliters of the extract solution was added into separate test tubes and ten mature brine shrimps were added into each test tube. The test tubes were incubated at 30 °C for 24 h and counted the live shrimp under magnification. The number of deaths of shrimps was plotted against extract concentration and the LD_50_ value was estimated [13]. Potassium dichromate dissolved in artificial seawater at concentrations ranging from 125 to 1000 μg/ml was used as positive controls of the experiment. Each experiment was performed in was performed in triplicates.

## Results

### In vitro α-amylase inhibitory studies

**Fig. 1:**
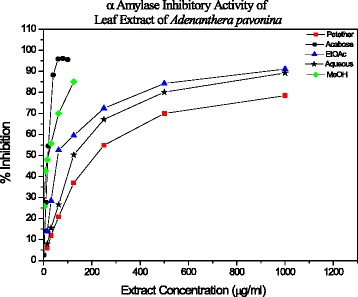
Enzyme inhibition (%) as a function of *Adenanthera pavonina* extract concentration

Evaluating the plot of % α-amylase inhibition as a function of extract concentrations (Fig. 1), the IC_50_ values were calculated (Table 1). The crude MeOH extract exhibited the lowest IC_50_ of 16.16 ± 2.23 μg/ml and the IC_50_ values of EtOAc, petroleum ether, and the water extracts were 59.93 ± 0.25, 145.49 ± 4.86 and 214.85 ± 9.72 μg/mL respectively. The standard positive control Acarbose showed an IC_50_ of 18.63 ± 1.21 μg/ml. In this study, significant differences were found between the IC_50_ values of all extracts (*P* =2.23 × 10^−11^, one-way ANOVA). However, the difference between the IC_50_ of Acarbose and the methanol extract did not show a significant difference (*P* = 0.175, one-way ANOVA).

**Table 1:** Total Phenolic Content (as Gallic acid equivalents), IC_50_ (μg/ml) of DPPH scavenging activity and IC_50_ (μg/ml) of α amylase inhibitory activity of *Adenanthera pavonina* leaf extracts

Extract	Total Phenolic Content (Gallic acid equivalents mg/g extract)	DPPH Scavenging Activity (IC_50_ (μg/ml)	α-amylase inhibitory activity IC_50_ (μg/ml)
Crude Methanol	9.73 ± 0.12	423.62 ± 2.45	16.16 ± 2.23
Ethyl Acetate	34. 62 ± 1.14	249.92 ± 3.35	59.93 ± 0.25
Petroleum Ether	13.46 ± 0.11	751.66 ± 4.91	145.49 ± 4.86
Aqueous	2.88 ± 0.23	>1000	214.85 ± 9.72

### Antioxidant activity

#### Total phenolic content

**Fig. 2:**
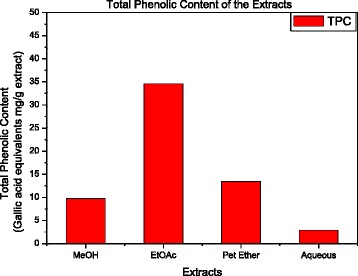
Total phenolic content as Gallic acid equivalents in extracts of *Adenanthera pavonina* extracts

The total phenolic content (TPC) is expressed as Gallic acid equivalents in mg/g of extract (Table 1, Fig. 2). The crude MeOH extract of *A. pavonia* contained 9.73 ± 0.12 mg Gallic acid equivalents/g of extract and the EtOAc aqueous and the petroleum ether extract showed a phenolic content of 34. 62 ± 1.14, 13.46 ± 0.11 and 2.88 ± 0.23 mg of Gallic acid equivalents per gram extract respectively.

### DPPH radical scavenging activity

**Fig. 3:**
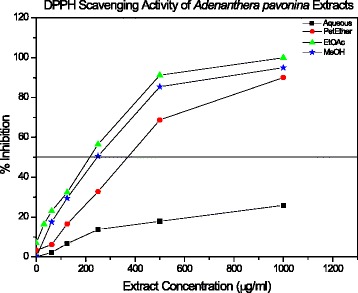
The plot of DPPH radical scavenging activity as a function of *Adenanthera pavonina* extract concentrations

The DPPH radical scavenging activities of the *A. pavonina* extracts showed IC _50_ value of 751.66 ± 4.91, 423.62 ± 2.45 and 249.92 ± 3.35 μg/ml for the extracts of petroleum ether crude methanol and ethyl acetate respectively (Fig. 3, Table 1). The best activity was observed in the EtOAc fraction with an IC_50_ value of 249.92 ± 3.35 μg/ml and IC_50_ of the aqueous extract was not determined, as it was greater than 1 mg/ml. The % inhibition of the aqueous extract at 1 mg/ml was only 25.87 ± 1.75%. Significant differences were found between the IC_50_ values of all extracts for the DPPH scavenging activity (*P* =0.00019, one-way ANOVA).

### Brine shrimp cytotoxicity assay

LD_50_ values lower than 1000 μg/ml is considered to be cytotoxic in the toxicity evaluation of plant extracts using brine shrimp assay [13]. The LD_50_ value of the crude methanol extract of *A. pavonina* leaves was estimated to be 1963.936 μg/ml indicating the absence of cytotoxic compounds in the extract.

## Discussion

The trend in the screening of medicinal plants for antidiabetic activity has increased, as it is important to discover novel effective drugs for the disease. However, the WHO has recommended daily exercise and healthy food intake as an effective method of controlling the diabetes type II [1]. Therefore promoting the urban population for a healthy living style with the use of greens that possess anti-diabetic activity and antioxidant activity in their daily diet would be one of the cost-effective ways of controlling the disease. Though seed and leaf extracts have exhibited hypoglycemic and antihyperglycemic activity as tested on rat models this study would be the first report on detailed α- amylase inhibitory activity study to the best of our knowledge.

The α-amylase inhibitory studies performed demonstrated that the extracts of *A.pavonina* (crude MeOH, EtOAc, pet-ether and water) had significant inhibitory potentials. The IC_50_ value (16.16 ± 2.30 μg/ml) of methanol extracts is almost similar to that of Acarbose (18.63 ± 1.21 μg/ml) a widely used and marketed anti-diabetic drug. These α- amylase inhibitors are also called as starch blockers as they prevent or slows the absorption of starch into the body mainly by blocking the hydrolysis of 1,4-glycosidic linkages of starch and other oligosaccharides into maltose, maltriose and other simple sugars [14]. The α amylase inhibitory activity in methanol extract is most likely to be due to polar compounds and is worth investigating further and isolating pure active compounds.

It is predicted that diabetic complications occur as a result of the oxidative stress due to the formation of free radicals with the glucose oxidation and the subsequent oxidative degradation of glycated proteins [15]. Therefore the use of antioxidants along with anti-diabetic drugs are frequently recommended to avoid such complications. The antioxidant activities measured by DPPH scavenging properties and the total phenolic content of the *A. pavonina* leaf extracts reveals all extracts does possess mild antioxidant properties with ethyl acetate fraction exhibiting the best DPPH scavenging property (IC_50_ of 249.92 ± 3.35 μg/ml) and with the highest total phenolic content of 34. 62 ± 1.14 (Gallic acid equivalents mg/g extract). The preliminary investigation carried out on cytotoxicity using brine shrimps show that the methanol leaf extract does not carry toxic compounds as the LD_50_ value was higher than 1 mg/ml (1963.936 μg/ml).

Hence the above results suggest that the leaf extracts of *A. pavonina* could be greatly beneficial in reducing the absorption of starch into the body also can be effectively used in ayurvedic treatments. Since leaves of *A. pavonina* is used mostly among the rural population as a green vegetable use of this leafy vegetable can be promoted among the urban population for its health benefits especially in the management of diabetics.

## Conclusion

According to the result of the study on the leaf extracts of *Adenanthera pavonina* exhibit α-amylase inhibitory activity with remarkable activity in the crude methanolic extract. Hence leaves of *Adenanthera pavonina* has the potential to be used as a green vegetable and also be used in ayurvedic decoctions in controlling and treatment of Type II diabetes mellitus. Furthermore, this study has opened opportunities for future research in searching for novel effective drugs for diabetics that possess both antioxidant activity and anti-diabetic activity.
